# Blood pressure and body mass index in an ethnically diverse sample of adolescents in Paramaribo, Suriname

**DOI:** 10.1186/1471-2261-9-19

**Published:** 2009-05-21

**Authors:** Charles Agyemang, Eline Oudeman, Wilco Zijlmans, Johannes Wendte, Karien Stronks

**Affiliations:** 1 Department of Social Medicine, Academic Medical Centre, Amsterdam, The Netherlands; 2 Department of Pediatrics, Diakonessen Hospital, Paramaribo, Suriname

## Abstract

**Background:**

High blood pressure (BP) is now an important public health problem in non-industrialised countries. The limited evidence suggests ethnic inequalities in BP in adults in some non-industrialised countries. However, it is unclear whether these ethnic inequalities in BP patterns in adults reflect on adolescents. Hence, we assessed ethnic differences in BP, and the association of BP with body mass index (BMI) among adolescents aged 12–17 years in Paramaribo, Suriname.

**Methods:**

Cross-sectional study with anthropometric and blood pressure measurements. A random sample of 855 adolescents (167 Hindustanis, 169 Creoles, 128 Javanese, 91 Maroons and 300 mixed-ethnicities) were studied. Ethnicity was based on self-reported ethnic origin.

**Results:**

Among boys, Maroons had a lower age- and height-adjusted systolic BP than Creoles, and a lower diastolic BP than other ethnic groups. However, after further adjustment for BMI, only diastolic BP in Maroons was significantly lower than in Javanese (67.1 versus 70.9 mmHg). Creole boys had a lower diastolic BP than Hindustani (67.3 versus 70.2 mmHg) and Javanese boys after adjustment for age, height and BMI. Among girls, there were no significant differences in systolic BP between the ethnic groups. Maroon girls, however, had a lower diastolic BP (65.6 mmHg) than Hindustani (69.1 mmHg), Javanese (71.2 mmHg) and Mixed-ethnic (68.3 mmHg) girls, but only after differences in BMI had been adjusted for. Javanese had a higher diastolic BP than Creoles (71.2 versus 66.8 mmHg) and Mixed-ethnicity girls. BMI was positively associated with BP in all the ethnic groups, except for diastolic BP in Maroon girls.

**Conclusion:**

The study findings indicate higher mean BP levels among Javanese and Hindustani adolescents compared with their African descent peers. These findings contrast the relatively low BP reported in Javanese and Hindustani adult populations in Suriname and underscore the need for public health measures early in life to prevent high BP and its sequelae in later life.

## Background

The increasing prevalence of cardiovascular diseases (CVD) is putting a tremendous pressure on already overburdened resources in non-industrialised countries. [1] High blood pressure (BP) is a leading cause of CVD. [2] The rising prevalence of hypertension in non-industrialised countries reflects well on the high prevalence of CVD. [2]

In children, BP tracking patterns confirm that persistent BP increase may be related to hypertension in adulthood. [3-5] Increased BP in childhood has also been linked with left ventricular hypertrophy. [6] Consequently, in most industrialised countries assessment and management of BP in childhood is strongly recommended to promote improved cardiovascular health in adulthood. [7] However, in some non-industrialised countries, BP data on children and adolescents are very scarce. In Suriname, for example, there is no published data on BP in children and adolescents. Information on BP in adults in Suriname is also very limited. A report from II PAHO-DOTA Workshop on Quality of Diabetes Care in 2003 indicates that hypertension is a major public health burden with prevalence rates ranging from 24% in Javanese to as high as 33% in African-Surinamese in Suriname. [8] The annual report of the Regional Health Service in 2000 also showed that hypertension care alone accounted for 15% of the total number of consultations. [8] This reflected the mortality data with CVD being the leading cause of death in Suriname.

Suriname's population is made up of several ethnic groups. As in industrialised countries, [9-12] the limited data in Suriname suggest ethnic inequalities in BP and hypertension in adults. [8] However, it is unclear whether these BP patterns in adults reflect on adolescents. In some industrialised countries such as the UK, ethnic differences in BP in adults [9-12] do not correspond with children's and adolescents' BP patterns. [13,14] In addition, the prevalence of overweight and obesity in children and adolescent have increased dramatically over the past few decades. This may have an impact on BP. In the United States of America, for example, overweight children have been shown to be 2–4 times more likely than non-overweight children to have high BP. [15-17] However, information on the relationship between body sizes and BP among different ethnic groups in non industrialised countries is limited. There is an urgent need for research in children and adolescents so that appropriate cost-effective interventions can be introduced early in life to prevent the burden of CVD in adulthood. [18,19] The main objective of this study was to assess BP patterns, and to determine the association of BP with BMI among adolescents from different ethnic backgrounds in Paramaribo, Suriname. We hypothesised that the BP patterns in adults would reflect adolescents' BP patterns in various ethnic groups in Suriname.

## Methods

### Study area

**Figure 1:**
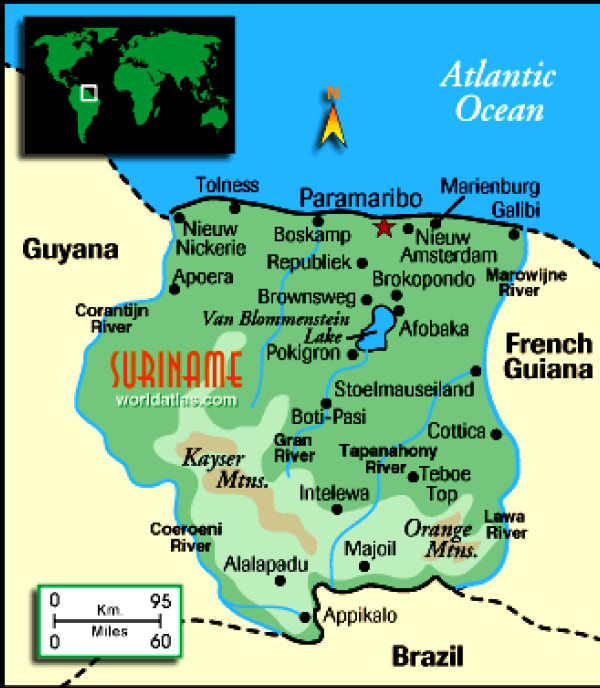
**Map of Suriname**.

Suriname is located in Northern South America. It borders French-Guyana to the east, British-Guyana to the west, Brazil to the south and the Atlantic Ocean to the north (Figure 1). It has a total area of 163,820 square kilometres. According to the World Factbook, the total population in 2007 was about 449,000 people. The life expectancy was 66 years for men and 71 years for women. Suriname's population is made up of several ethnic groups: Hindustani, Creoles, Javanese, Maroons, Amerindian, Chinese, White and other. About 28% of the population is under 15 years of age (ca. 132,000). Data for this study were collected between March and June 2007 in Paramaribo, the capital city of Suriname. The vast majority of people (about 90%) live in Paramaribo and its surroundings.

### Study design

Data were collected in nine schools among healthy adolescents between the ages of 12 to17 years. All schools were randomly selected from the 103 government schools' list. The schools were visited prior to data collection to obtain permission from the relevant school principals as well as from the pupils. After agreeing to this, participants were advised not to smoke, drink alcohol or take vigorous exercise during the 30 minutes preceding the BP measurement. Data collection took place during normal school hours. Verbal informed consent was sought from each participant before measurements were taken. None of the children refused to participate in this study. Forty three children were excluded because they were below 12 years old, and one child was excluded because she reported suffering from severe heart disease. The Ethical Board of the Ministry of Education in Suriname approved the study protocols.

### Measurements

Height was measured without shoes with a measuring tape to the nearest 0.01 metre. Weight was measured to the nearest 0.1 kg after removal of shoes, jackets, heavy clothing and pocket contents (using an Electronic Korona Profirmed scale). BMI was calculated as weight divided by height squared (kg/m^2^). Overweight and obesity were defined using the sex- and age-specific BMI criteria of the International Obesity Task Force. [20] BP was measured in the morning with a validated oscillometric automated digital BP device (Omron M-5, Japan). Using appropriate cuff sizes, three readings with one minute interval were taken on the right arm in a seated position after at least five minutes rest. The mean of the last two readings was used for analysis. Sex-, age- and height-specific percentile levels were defined using US normative BP tables for children and adolescents. [21] High BP was defined as systolic BP and/or diastolic BP ≥ 95th percentile. [21]

In addition to the physical measurements, participants were asked to complete a short questionnaire including questions on age, sex, ethnic background and physical activity. The ethnicity of these groups was identified using self-reported ethnicity. Hindustanis originate from India (South Asia). Javanese originate from Indonesia (East Asia). Creoles are descendants of West Africans who live in Paramaribo. The Maroons are descendants of West Africans who fled the colonial Dutch forced labour plantations in Suriname and established independent communities in the interior rainforests. They have retained a distinctive identity based on their West African origins. There are no secondary schools in their villages, so they attend schools in Paramaribo. Because of the important differences between these two West African descent groups in terms of lifestyle and history, we have separated these groups. Physical activity was based on the frequency of leisure physical exercise per week outside of school. The same trained final year medical student made anthropometric and blood pressure measurements in all schools.

### Data analysis

Age specific mean systolic BP and diastolic BP levels were determined for boys and girls. Multivariate linear regression analysis enabled age, height and BMI adjusted comparisons of systolic BP and diastolic BP levels to be made between different ethnic groups. Multiple linear regression analyses were performed separately for each ethnic group to assess the relationship between BMI and BP adjusted for other factors. All statistical tests were two-tailed and *P*-value < 0.05 was considered statistically significant. All statistical analyses were performed using SPSS for Windows version 14.2 (SPSS Inc. Chicago, USA) and STATA 9.2 (Stata Corp, College Station, Texas).

## Results

Table 1 shows the characteristics of the study population by sex and ethnicity. Javanese boys and girls were shorter than all the ethnic groups (P < 0.05). Maroon boys had a lower BMI than Creole boys (*P *= 0.03) and Mixed-ethnicity boys (*P *= 0.05). Maroon boys were more likely than other boys to exercise 5–7 days/week. Creole girls were taller than all the ethnic groups (*P *< 0.001). Maroon girls had a higher BMI than Hindustani girls (*P *= 0.03) and Javanese (*P *< 0.01) girls.

**Table 1:** Characteristics of the study population by ethnicity and sex

	Hindustani	Creole	Javanese	Maroon	Mixed	p-value
**Boys**	**(n = 86)**	**(n = 85)**	**(n = 60)**	**(n = 41)**	**(n = 117)**	
						
Age (y)	14.7 (1.5)	15.1 (1.2)	15.0 (1.2)	15.3 (1.2)	14.7 (1.4)	0.01
Heart rate	83.6 (12.5)	79.1 12.8)	82.3 (12.9)	81.0 (12.1)	79.1 (11.5)	0.06
Height (cm)	168.8 (9.6)	169.4 (9.5)	165.7 (8.7)	169.8 (8.3)	169.7 (8.4)	0.06
Weight (Kg)	58.1 (18.0)	60.6 (13.0)	56.1 (13.6)	56.0 (10.1)	60.0 (13.1)	0.20
BMI (Kg/m^2^)	20.1 (4.9)	21.0 (3.7)	20.2 (4.0)	19.3 (2.8)	20.7 (3.8)	0.18
Overweight/obesity, %	24.4	21.2	18.3	7.3	18.8	0.24
Exercise ≥5–7 days/week, %	18.6	28.2	20.0	43.9	29.1	0.03
						
**Girls**	**(n = 81)**	**(n = 84)**	**(n = 68)**	**(n = 50)**	**(n = 183)**	
						
Age (y)	14.5 (1.5)	14.7 (1.3)	14.4 (1.4)	15.0 (1.3)	14.8 (1.3)	0.11
Heart rate	87.4 (11.5)	86.4 (13.2)	87.0 (13.1)	84.4 (13.6)	82.6 (12.0)	0.02
Height (cm)	160.1 (5.7)	164.4 (7.0)	157.8 (6.1)	160.6 (5.0)	161.4 (6.3)	0.001
Weight (Kg)	51.1 (12.3)	56.4 (11.4)	48.2 (8.1)	55.0 (10.9)	53.0 (10.1)	0.001
BMI (Kg/m^2^)	19.5 (4.0)	20.8 (3.8)	19.3 (3.0)	21.3 (4.1)	20.3 (3.5)	0.02
Overweight/obesity, %	13.6	21.4	7.4	20.0	13.7	0.12
Exercise ≥5–7 days/week, %	7.4	6.0	4.4	4.0	8.2	0.74

Results are means (SD) and %; BMI, Body mass index

### Blood pressure levels

**Figure 2:**
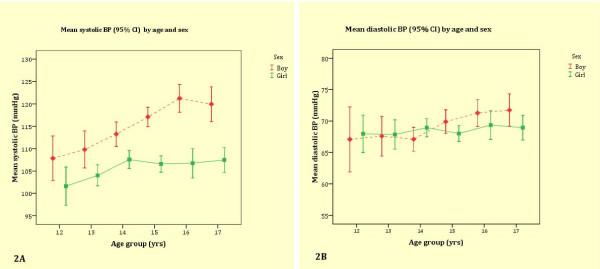
**Mean systolic and diastolic BP by age and sex**.

**Figure 3:**
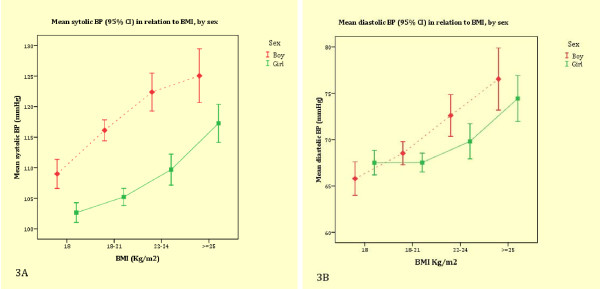
**Mean systolic and diastolic BP in relation to BMI by sex**.

The mean systolic BP and diastolic BP increased with age in both boys and girls (Figure 2a and 2b). The mean systolic BP levels were higher in boys than in girls in all age groups (Figure 2a). The sex differences in diastolic BP were, however, less pronounced (Figure 2b). BMI was associated with systolic BP and diastolic BP in both boys and girls (Figure 3a and 3b). Table 2 shows the relationships between BMI and systolic BP, and diastolic BP in each ethnic group. BMI was independently associated with systolic BP and diastolic BP in all ethnic groups except for diastolic BP in Maroons.

**Table 2:** Multiple regression analysis of factors associated with SBP and DBP for each ethnicity

		**Systolic blood pressure**			**Diastolic blood pressure**		
		*β*	(95% CI)	p-value	*β*	(95% CI)	p-value
Hindustani							
	BMI (Kg/m^2^)	1.27	(0.93, 1.60)	.000	0.79	(0.56, 1.02)	.000
	Girls	-10.76	(-13.75, -7.77)	.000	-1.90	(-3.99, 0.19)	.075
	Age	0.55	(-0.46, 1.56)	.285	0.73	(0.02, 1.43)	.043
	Heart rate	0.27	(0.14, 0.39)	.000	0.17	(0.08, 0.26)	.000
	Exercise ≥5–7 days/week	-4.65	(-9.02, -0.28)	.037	-2.98	(-6.03, 0.07)	.056
Creole							
	BMI (Kg/m^2^)	1.21	(0.75, 1.66)	.000	0.44	(0.14, 0.73)	.004
	Girls	-11.05	(-14.61, -7.50)	.000	-2.34	(-4.67, -0.01)	.049
	Age	1.84	(0.46, 3.22)	.009	0.50	(-0.40, 1.41)	.272
	Heart rate	0.19	(0.06, 0.32)	.005	0.22	(0.14, 0.31)	.000
	Exercise ≥5–7 days/week	-6.06	(-10.76, -1.36)	.012	-2.70	(-5.78, 0.38)	.085
Javanese							
	BMI (Kg/m^2^)	1.21	(0.67, 1.75)	.000	1.14	(0.75, 1.52)	.000
	Girls	-10.65	(-14.48, -6.82)	.000	-0.72	(-3.43, 2.00)	.602
	Age	0.94	(-0.43, 2.30)	.178	-0.02	(-0.99, 0.94)	.961
	Heart rate	0.29	(0.15, 0.43)	.000	0.23	(0.13, 0.33)	.000
	Exercise ≥5–7 days/week	-6.55	(-12.58, -0.51)	.034	-4.28	(-8.56, -0.01)	.050
Maroon							
	BMI (Kg/m^2^)	0.92	(0.09, 1.75)	.030	0.23	(-0.32, 0.79)	.411
	Girls	-8.20	(-15.10, -1.29)	.021	-0.71	(-5.32, 3.90)	.760
	Age	2.10	(-0.22, 4.43)	.075	1.75	(0.20, 3.30)	.028
	Heart rate	0.16	(-0.07, 0.38)	.167	0.21	(0.06, 0.36)	.006
	Exercise ≥5–7 days/week	-0.56	(-8.42, 7.29)	.887	0.77	(-4.48, 6.02)	.771
Mixed							
	BMI (Kg/m^2^)	1.29	(0.94, 1.63)	.000	0.84	(0.57, 1.12)	.000
	Girls	-11.04	(-13.66, -8.43)	.000	-2.11	(-4.25, 0.02)	.052
	Age	1.08	(0.13, 2.02)	.025	0.22	(-0.55, 0.99)	.579
	Heart rate	0.13	(0.03, 0.23)	.013	0.17	(0.09, 0.26)	.000
	Exercise ≥5–7 days/week	0.23	(-3.18, 3.65)	.894	-2.73	(-5.52, 0.06)	.055

BMI, body mass index, CI, confidence intervals

**Figure 4:**
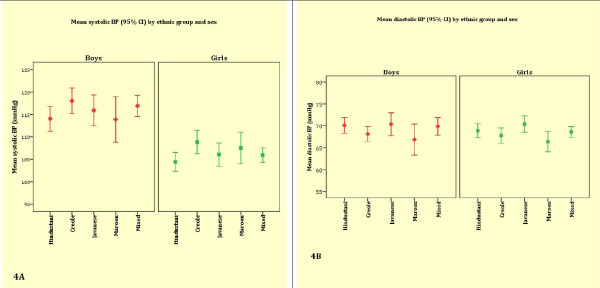
**Mean systolic and diastolic BP by ethnicity and sex**.

Figure 4a and 4b show mean systolic BP and diastolic BP by ethnic group and sex. After adjustment for age and height, Maroon boys had a significantly lower mean systolic BP than Creole boys and a lower mean diastolic BP than all the other ethnic groups (Table 3). Further adjustment for BMI abolished the significant mean systolic and diastolic BP differences between Maroon boys and other ethnic groups except for the higher mean diastolic BP in Javanese boys (*P *= 0.04). Creole boys had a relatively low age- and height-adjusted mean diastolic BP compared to other boys. After further adjustment for BMI, the differences became more pronounced between Creole and Hindustani boys (P = 0.04) and Javanese boys (P = 0.02).

**Table 3:** Adjusted mean systolic and diastolic blood pressure levels by ethnicity and sex

	**Systolic blood pressure [95% CI]**		**Diastolic blood pressure [95% CI]**	
	**Model I**	**Model II**	**Model I**	**Model II**
				
**Boys**				
				
Hindustani	114.2 (111.6–116.8)	114.4 (112.0–116.9)	70.0 (68.1–72.0)	70.2 (68.3–72.1)
Creole	117.2 (114.5–119.8)	116.7 (114.2–119.1)	67.6 (65.4–69.9)	67.3 (65.4–69.2)
Javanese	117.3 (114.1–120.4)	117.3 (114.3–120.2)	70.9 (68.5–73.3)	70.9 (68.6–73.2)
Maroon	112.5 (108.8–116.3)	114.0 (110.5–117.6)	66.1 (63.2–69.0)	67.1 (64.3–69.9)
Mixed-ethnicity	116.5 (114.3–118.8)	116.2 (114.1–118.3)	69.6 (67.9–71.4)	69.4 (67.8–71.0)
				
**Girls**				
				
Hindustani	104.5 (102.1–106.9)	105.0 (102.8–107.2)	68.8 (67.1–70.6)	69.1 (67.4–70.7)
Creole	107.9 (105.4–110.3)	107.2 (105.0–109.4)	67.2 (65.4–68.9)	66.8 (65.1–68.5)
Javanese	106.7 (104.1–109.4)	107.7 (105.0–110.2)	70.6 (68.7–72.6)	71.2 (69.3–73.0)
Maroon	107.3 (104.2–110.4)	106.2 (103.4–109.0)	66.1 (64.0–68.4)	65.6 (63.4–67.9)
Mixed-ethnicity	105.6 (103.9–107.1)	105.6 (104.1–107.1)	68.3 (67.2–69.5)	68.3 (67.2–69.4)

Model I (adjusted for age and height) model II (model I plus BMI); CI, confidence intervals

Among girls, Javanese had a significantly higher age- and height-adjusted mean diastolic BP than all other ethnic groups except for Hindustani girls. The differences persisted after further adjustment for age, height and BMI (*P *< 0.01). Maroon girls had a significantly lower mean diastolic BP than all ethnic groups, except for Creole girls, but only after differences in BMI had been adjusted for. There were no significant differences in mean systolic BP between the ethnic groups.

**Figure 5:**
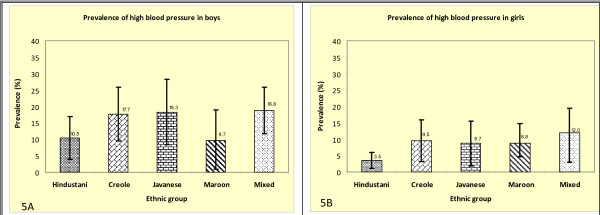
**Prevalence of high BP in boys and girls**.

Maroon boys and Hindustani boys and girls had a relatively low prevalence of high BP compared with other ethnic groups although the differences were not statistically significant (Figure 5a and 5b).

## Discussion

As far as we know, this is the first study on BP patterns amongst adolescents in Suriname, and one of the few studies in adolescents comparing different ethnic groups in a non-industrialised country. Maroon boys had a lower BP than all ethnic groups including Creoles with similar West African descent. The lower BP in Maroon boys as compared to other ethnic groups was accounted for by their lower BMI. This might reflect differences in environmental factors such as lifestyle between Maroons and other ethnic groups. Maroon adolescents moved from the Suriname interior to Paramaribo to continue their education. Most Maroon people in Suriname interior still live traditional African lifestyles with female subsistence horticulture and male hunting and fishing. [22] Most Maroon villages are located along the rivers of the interior of Suriname and access is heavily dependent on canoes and other watercraft. [22] This population is therefore isolated and is less exposed to unhealthy urban lifestyles such as excessive consumption of energy-dense foods and frequent use of automobiles [23,24] in urban Paramaribo. It is possible they were still benefiting from their earlier exposure to the traditional lifestyles, which have been suggested to be protective against high BP. [25] Maroon girls, however, had relatively high prevalence of overweight and obesity compared with other ethnic groups. The lower BP in Maroon girls became apparent only after further adjustment for BMI. It is possible that they have adopted different lifestyles such as excessive energy intake and decreasing physical activity once in unban Paramaribo, which may contribute to their rapid increase in BMI and subsequently higher BP. In this study, only 4% of Maroon girls reported ≥ 5–7 days/week physical activity outside of school. These findings clearly indicate the need to promote physical activity among this group in Suriname.

Studies in adults have consistently shown higher BP levels in African descent people than in other ethnic groups. [5,9-11] Studies in Suriname and the Netherlands, for example, showed higher BP levels in African-Surinamese than in other ethnic groups. [8,9] In this present study, however, African descent adolescents in Suriname (both Creoles and Maroons) had lower mean diastolic BP levels than other ethnic groups. The lack of higher BP levels in African descent adolescents in Suriname is consistent with the findings in the UK. Studies in the UK show higher BP levels in African descent adults than in other ethnic groups. [10,26] By contrast, in adolescents, BP levels were either lower or similar in African descent youth than in other ethnic groups. [13,14] A recent study, for example, found that BP in ethnic minority adolescents was generally lower than in White adolescents except for diastolic BP among Indian girls in the UK. [13]

The explanations for the different patterns of BP in adolescents and adulthood among African descent populations are unclear and require a cohort study to unravel the possible mechanisms underlying these differences. These observations suggest that environmental factors may be very important. The higher BP among Javanese adolescents was unexpected given the lower BP reported among the Javanese adult population in Suriname. The reason for the higher mean BP among Javanese adolescents is unclear. One possible explanation may relate to generational differences or changes in lifestyles. This requires further study. Left unchecked, the comparatively high BP among Javanese adolescents may abolish or reverse the current lower BP advantage enjoyed by the Javanese adult population in Suriname. These observations clearly indicate the need for early intervention in adolescents for preventing high BP in later life. [13]

The relationship between BMI and BP is well established in children and adolescents. [15-17] The strong and independent relationship between BMI and BP in our present study is consistent with previous findings. Although the mechanisms by which BMI may lead to high BP are not well understood, it is now generally recognised that high BMI significantly increases the risk of high BP. [27] Sinaiko and colleagues' prospective study showed that increases in BMI in early life were significantly related to an increased risk of high BP and other CVD in adulthood. [27] Our findings clearly indicate the need to prevent the increasing prevalence of overweight and obesity especially in Maroon girls early in life to prevent future sequelae of overweight/obesity related diseases.

Our study has limitations. As in many epidemiological studies, our BP level was based on an average of two measurements at a single visit. A more precise estimate of BP level would be obtained by multiple measurements obtained during several visits. Also, evidence suggests that during puberty BP increases more rapidly, with a significant gender difference in the age of onset. [28] In the present study, pubertal status was not assessed and this may affect the study conclusions. Nevertheless, in the recent UK study, late puberty was not associated with high BP in ethnic minority groups. [13] Another possible limitation is the combined mixed-ethnicities due to the small study samples. It is possible that BP patterns differ among these different ethnic groups. [29] Future studies should assess this possibility. In addition, social circumstances between the groups were not assessed, which might also affect our study conclusions. Despite these limitations, our present findings provide very important insights into ethnic differences in BP in adolescents in non-industrialised setting.

## Conclusion

The study findings indicate a higher mean BP among Hindustani boys and Javanese boys and girls whereas in the adult population these groups have lower mean BP levels in Suriname. BMI was positively related to BP in all the ethnic groups. These observations underscore the urgent need for public health measures early in life to prevent high BP and its sequelae in later life. BP reductions in adolescents can be achieved by weight loss through reducing excessive energy intake [30] and increasing physical activity strategies. [31] These cost-effective measures may lead to an important reduction in BP in adolescents thereby sparing the next generation from hypertension related complications [32].

## Competing interests

The authors declare that they have no competing interests.

## Authors' contributions

All were responsible for study concept and design. EO and WJ were responsible for data collection. CA, JFW, EO and KS were responsible for analysis and interpretation of data. CA drafted the manuscript and all were involved in critical revision of the manuscript. All authors read and approved the final manuscript.

## Pre-publication history

The pre-publication history for this paper can be accessed here:


